# The Prevalence of Dermoscopy Use Among Dermatology Residents in Riyadh, Saudi Arabia: Cross-Sectional Study

**DOI:** 10.2196/63861

**Published:** 2025-01-23

**Authors:** Abdullah Almeziny, Rahaf Almutairi, Amal Altamimi, Khloud Alshehri, Latifah Almehaideb, Asem Shadid, Mohammed Al Mashali

**Affiliations:** 1 College of Medicine, Al-Imam Muhammad Ibn Saud Islamic University, Riyadh, 13317, Saudi Arabia, 966 538699522; 2 Department of Dermatology, King Fahad Medical City, Riyadh, Saudi Arabia; 3 Department of Dermatology, College of Medicine, Imam Mohammad Ibn Saud Islamic University, Riyadh, Saudi Arabia

**Keywords:** dermoscopy, Saudi Arabia, questionnaire, skin lesion, noninvasive, cross-sectional study, dermatology

## Abstract

**Background:**

Dermoscopy is a noninvasive technology used to examine the skin’s invisible microstructures in dermatological practice and is gaining prominence as a crucial tool. Dermoscopy is an evidence-based practice used to enhance the early detection of skin malignancies and to help distinguish between various skin conditions, including pigmented and nonpigmented skin malignancies. Currently, the vast majority of global guidelines for skin cancer recommend dermoscopy as a critical component. Dermoscopy use is increasing worldwide, but to date, no study has documented the attitudes toward and use of dermoscopy among future dermatologists in Saudi Arabia.

**Objective:**

We aimed to determine the proportion of dermatology residents in Riyadh who use dermoscopy in their clinical practice; identify factors influencing the use of dermoscopy, such as availability of equipment, training, and the perceived importance of dermoscopy in clinical practice; explore barriers to dermoscopy use, including the lack of access to necessary resources (eg, dermoscopes) and insufficient training; and provide insights into the adoption and integration of dermoscopy into dermatology training and clinical practice in Saudi Arabia.

**Methods:**

In January 2024, a validated and published questionnaire was modified to meet research requirements and was sent to all registered dermatology residents in the The Saudi Board of Dermatology and Venereology Program.

**Results:**

In total, 63 dermatology residents in Riyadh, Saudi Arabia, completed the web-based questionnaire (response rate=87.5%). The sample was predominantly female (n=34, 54.0%), with the majority (n=53, 84.1%) aged between 26 and 30 years. A notable proportion of participants (n=22, 34.9%) were in their final year of residency. Over half of the participants (n=34, 54.0%) owned a dermoscope, and a substantial number of them (n=23, 36.5%) reported conducting 21-30 clinic consultations per month on average. More than half of the participants (n=36, 57.1%) had received dermoscopy training, and 16 (36.4%) had used dermoscopy for 2 years. Additionally, most participants (n=20, 45.5%) had used nonpolarized immersion-contact dermoscopy, while 19 (43.2%) had used polarized light dermoscopy. Furthermore, the majority (n=22, 50.0%) used dermoscopy in fewer than 10% of cases involving patients with inflammatory skin lesions. Statistical analysis revealed significant associations between the participants’ ages (*P*=.003), residency levels (*P*=.001), and practice centers and the use of dermoscopy (*P*=.004).

**Conclusions:**

Dermoscopy has been widely adopted by dermatology residents in their daily clinical practice due to its benefits in early detection and diagnosis of skin diseases. However, the overall extent of dermoscopy use within the dermatology community remains unclear, highlighting the need for further education. In Saudi Arabia, the key factors influencing dermoscopy use include residents’ ages, residency levels, and practice centers. Younger dermatologists have expressed strong interest in improving their dermoscopy knowledge and skills. Expanding access to dermoscopy equipment and providing training during residency could further promote its use across the country.

## Introduction

Dermoscopy is a noninvasive technology used to examine the skin’s invisible microstructures in dermatological practice [1]. It is an established technique for analyzing skin lesions, with its origins tracing back to the 17th century when Kohlhaus used a microscope to study nail matrix vessels [1-3]. However, dermoscopy did not gain widespread use until the 20th century, when Goldman developed a portable microscope capable of magnifying up to 10 times more than the naked eye [1-6]. Historically, dermoscopy has been used to diagnose pigmented lesions such as naevi, melanomas, and pigmented basal cell carcinomas [7].

Since the 1990s, it has been used to diagnose other dermatological disorders, including infections such as scabies, inflammatory lesions, and hair and nail-fold alterations, and it is also used to track lesions’ progress or reactions to topical treatments such as imiquimod or 5-fluorouracil [8-11]. The evidence-based practice associated with dermoscopy use improves the diagnostic accuracy for skin cancer, reduces unnecessary biopsies of benign lesions, increases survival rates, and improves the early identification of skin malignancies [12-14]. A 2002 meta-analysis of 27 studies revealed that dermoscopy increases experienced physicians’ diagnostic accuracy for melanoma compared to unaided inspection [1]. Moreover, it helps distinguish between various kinds of pigmented and nonpigmented skin malignancies in vivo, and it is significant in differentiating between inflammatory and neoplastic lesions. Currently, the vast majority of worldwide guidelines for skin cancer recommend dermoscopy as a critical component of diagnosing and following up with patients facing an increased risk of this disease [15-20]. Dermoscopy is also increasingly accepted as a standard practice worldwide. Multiple studies have revealed that US dermatologists use dermoscopy. Indeed, 1555 out of 3238 (48%) American dermatologists surveyed in 2010 said they used dermoscopy [21]. Chamberlain’s study of dermoscopy revealed a 98% usage rate use in Australia [22,23], while 95% of dermatologists in France use the practice [24]. However, no study has documented the attitudes toward and use of dermoscopy among future dermatologists in Saudi Arabia. In this study, dermoscopy prevalence among dermatology residents in Riyadh was assessed alongside information sources and elements that influence its use, such as residency levels and the frequency of dermoscopy diagnoses.

## Methods

### Study Design

A cross-sectional study was conducted in Riyadh to determine the attitudes of dermatologists toward the dermoscopy. The convenience sampling technique was used in this study to recruit the study participants. The questionnaire tool was distributed in January 2024 through email registered at the Saudi Commission for Health Specialists.

The inclusion criterion for this study was to be a registered dermatology resident in Saudi boards in Riyadh. Any participants who did not meet this inclusion criterion was excluded from this study.

### Questionnaire Tool

This study adapted and used a questionnaire previously tested and found valid and reliable by Forsea et al [25]. The questionnaire comprises 2 sections: the first section collected information related to participants’ demographics (gender, age, and residency level), and in the second section, the future dermatologists who participated were asked about their perspectives about the utility of dermoscopy, their use patterns, their training experiences, and their self-reported confidence in dermoscopy diagnosis.

### Ethical Considerations

The study protocol was reviewed and approved by the Regional and institutional human medical biological research ethics committee of Al-Imam Muhammad Ibn Saud Islamic University (approval 735/2024). Participation in the study was entirely voluntary, and informed written consent was obtained from all participants before their involvement. The study was conducted in accordance with the principles of the declaration of Helsinki. All data collected were anonymized to ensure the privacy and confidentiality of the participants.

### Study Analysis

All research data were entered into a Microsoft Excel (version 16.0) spreadsheet. Data analysis was performed using SPSS (version 28; IBM Corp). Descriptive statistics were used in the statistical analysis; relative frequencies (and percentage values) were used to present categorical variables. The chi-square test was used to assess the association between categorical variables and dermoscopy use, with a 2-sided value of *P*<.05 considered statistically significant.

## Results

A total of 63 dermatology residents in Riyadh, Saudi Arabia, completed the web-based questionnaire, yielding an 87.5% response rate. More than half of the participants (n=34, 54.0%) were female, the majority (n=53, 84.1%) were aged between 26 and 30 years, and a considerable proportion (n=22, 34.9%) were in their fourth year of residency (Table 1).

**Table 1.** Participants’ (N=63) sociodemographic information.

Sociodemographic characteristics		Participants, n (%)
**Gender**		
	Female	34 (54.0)
	Male	29 (46.0)
**Age (years)**		
	20‐25	8 (12.7)
	26‐30	53 (84.1)
	31‐35	1 (1.6)
	36‐40	1 (1.6)
**Residency level**		
	Residency year 1	9 (14.3)
	Residency year 2	19 (30.2)
	Residency year 3	13 (20.6)
	Residency year 4	22 (34.9)

Table 2 (below) depicts practice characteristics, dermoscopy training, and dermoscopy use patterns among the participating dermatology residents in Riyadh, Saudi Arabia. More than half of the participants (n=34; 54.0%) owned a dermoscope, while a substantial number of them (n=23, 36.5%) offered an average of 21‐30 monthly clinic consultations. Most participants (n=57, 90.5%) presented an average number of 0-5 clinic consultations per month where they saw patients with cancer (all types). More than half of the participants (n=36, 57.1%) had received dermoscopy training. The majority of the participants (n=44, 69.8%) used dermoscopy, and a significant number of them had been inspired to do so by their colleagues (n=12, 27.3%) and mentors (n=9, 20.5%). The reported reasons for not using dermoscopy were its unavailability in an office (n=8, 42.1%) and a lack of training (n=6, 31.6%). Half of the participants (n=22, 50.0%) reported having used dermoscopy pictures in medical education, particularly in conferences, lectures, and academic activities. Most of the participants (n=17, 38.6%) had completed a rotation at King Saud University Medical City in 2023. A considerable proportion of participants (n=16, 36.4%) had used dermoscopy for 2 years; the majority used a nonpolarized immersion-contact dermoscope (n=20, 45.5%) or polarized-light dermoscope (n=19, 43.2%). Regarding their average practice, the majority of participants (n=15, 34.1%) reported using dermoscopy at least once per day. ABCD (Asymmetrical, Border, Color, Diameter) was reported to be the most common algorithm used by the majority of the participating dermatology residents (n=23, 52.3%) for the diagnosis of pigmented lesions.

**Table 2.** Dermoscopy practice characteristics, training, and use patterns.

Question and categories		Participants, n (%)
**Do you own a dermoscope?**		
	Yes, I do	34 (54.0)
	It is provided in the clinic	9 (14.3)
	No, I do not own one, nor is it provided	20 (31.7)
**What is your average number of monthly clinic consultations?**		
	0‐10	14 (22.2)
	11‐20	22 (34.9)
	21‐30	23 (36.5)
	31‐40	2 (3.2)
	More than 40	2 (3.2)
**What is the average number of monthly clinic consultations where you see patients with skin cancer (of all types)?**		
	0‐5	57 (90.5)
	6‐10	3 (4.8)
	11‐20	2 (3.2)
	More than 20	1 (1.6)
**Have you received dermoscopy training as part of your dermatology residency?**		
	Yes	36 (57.1)
	No	27 (42.9)
**Outside of your residency training, what type of dermoscopy training have you pursued?**		
	Academic activities provided by the residency program	21 (33.3)
	Dermoscopy course	5 (7.9)
	Web-based dermoscopy course	14 (22.2)
	Attended conferences or congresses	3 (4.8)
	Books or atlases	3 (4.8)
	A mentor or tutor	4 (6.3)
	No training	13 (20.6)
**Do you use dermoscopy?**		
	Yes	44 (69.8)
	No	19 (30.2)
**Which of the following made you consider using dermoscopy?**		
	A colleague	12 (27.3)
	A mentor	9 (20.5)
	Conference lectures	6 (13.6)
	Evidence-based practice	4 (9.1)
	Lectures provided by dermatology Saudi boards residency program	3 (6.8)
	A paid workshop	2 (4.5)
	Other	8 (18.2)
**If you do not use dermoscopy, please give the reason why not.**		
	A dermoscope is not available in my office	8 (42.1)
	I have not been trained in dermoscopy	6 (31.6)
	Other	5 (26.3)
**Have you used dermoscopy pictures in medical education?**		
	No, I have not used them	18 (40.9)
	Yes, in conferences, lectures, academic activities, etc	22 (50.0)
	Yes, in publications in articles or journals	4 (9.1)
	Other	20 (31.7)
**In the last year, where was your rotation?**		
	King Faisal Specialist Hospital	10 (22.7)
	King Saud University Medical City	17 (38.6)
	Ministry of National Guard Hospital	12 (27.3)
	Prince Sultan Military Medical City	5 (11.4)
**For how long have you been using dermoscopy?**		
	1 years	13 (29.5)
	2 years	16 (36.4)
	3 years	12 (27.3)
	4 years	3 (6.8)
**What type of dermoscope do you use?**		
	Nonpolarized immersion-contact dermoscope (contact with the skin and an interface liquid, eg, oil or alcohol)	20 (45.5)
	Polarized-light dermoscope	19 (43.2)
	Dermoscope with a digital camera	2 (4.5)
	Digital video dermoscopy system (eg, Fotofinder or Molemax)	3 (6.8)
**In your average practice, how often do you use dermoscopy?**		
	Less than once per month	5 (11.4)
	1‐4 times per month	13 (29.5)
	More than once per week	11 (25.0)
	At least once per day	15 (34.1)
**Which particular algorithm for the dermoscopic diagnosis of pigmented lesions do you regularly use?**		
	ABCD^a^ rule	23 (52.3)
	I do not systematically use any particular algorithm	10 (22.7)
	Menzies’s algorithm	1 (2.3)
	Pattern analysis	7 (15.9)
	Seven-point checklist	3 (6.8)

a ABCD: Asymmetrical, Border, Color, Diameter.

Table 3 illustrates clinical practices and the confidence in dermoscopy skills among the participating dermatology residents in Riyadh, Saudi Arabia. Our findings revealed that the majority of the participants (n=22, 50.0%) used dermoscopy in fewer than 10% of cases involving patients with inflammatory skin lesions. Moreover, a substantial proportion of participants (n=15,34.0%) used dermoscopy in more than 70% of cases involving the examination of pigmented skin tumors. Eleven (25.0%) participants used dermoscopy for <10% of their patients who were examined for nonpigmented skin tumors. Regarding the participants’ dermoscopy skills, the majority of them were somewhat confident in the assessment of nonpigmented skin tumors (n=26, 59.1%), inflammatory skin lesions (n=22, 50.0%), and pigmented skin tumors (n=19, 43.2%).

**Table 3.** Clinical dermoscopy practices and confidence in dermoscopy skills.

Category		Pigmented skin tumors, n (%)	Nonpigmented skin tumors, n (%)	Inflammatory skin lesions, n (%)
**When examining patients with the following disorders, in what percentage of cases do you use dermoscopy?**				
	<10% of cases	9 (20.5)	11 (25.0)	22 (50.0)
	11%‐30% of cases	8 (18.2)	8 (18.2)	10 (22.7)
	31%‐50% of cases	4 (9.1)	6 (13.6)	5 (11.4)
	51%‐70% of cases	8 (18.2)	10 (22.7)	3 (6.8)
	>70% of cases	15 (34.0)	9 (20.5)	2 (4.5)
**How confident are you in your dermoscopy skills for the assessment of the following types of lesions?**				
	Not confident	12 (27.3)	8 (18.2)	11 (25.0)
	Somewhat confident	19 (43.2)	26 (59.1)	22 (50.0)
	Confident	13 (29.5)	10 (22.7)	11 (25.0)

Table 4 illustrates the usefulness, advantages, and performance of dermoscopy. The vast majority of the participants (n=41, 93.2%) reported that dermoscopy was useful in diagnosing melanoma and following up on melanocytic lesions (n=39, 88.6%), diagnosing pigmented skin tumors (n=35, 79.5%), and diagnosing nonpigmented skin tumors (n=31, 70.5%). Regarding advantages, the majority of the participants agreed that dermoscopy use increases confidence in their clinical diagnoses (n=30, 68.2%), reduces unnecessary biopsies or excisions (n=27, 61.4%), and improves record-keeping (n=25, 56.8%). Weighing in on performance, more than half of the participants (n=30, 68.2%) reported that dermoscopy use increases the number of melanomas detected compared to naked-eye examinations. Additionally, the majority of participants (n=27, 61.4%) noted that the use of dermoscopy reduces the excision of benign lesions.

**Table 4.** Usefulness, advantages, and performance of dermoscopy use.

Category			Participants, n (%)
**Usefulness of dermoscopy**			
	**Diagnosis of melanoma**		
		Not useful	1 (2.3)
		Somewhat useful	2 (4.5)
		Useful	41 (93.2)
	**Follow-up on melanocytic lesions**		
		Not useful	0 (0)
		Somewhat useful	5 (11.4)
		Useful	39 (88.6)
	**Diagnosis of pigmented skin tumors**		
		Not useful	0 (0)
		Somewhat useful	9 (20.5)
		Useful	35 (79.5)
	**Diagnosis of nonpigmented skin tumors**		
		Not useful	1 (2.3)
		Somewhat useful	12 (27.3)
		Useful	31 (70.5)
	**Diagnosis of inflammatory skin lesions**		
		Not useful	3 (6.8)
		Somewhat useful	19 (43.2)
		Useful	22 (50.0)
	**Follow-up on nonmelanocytic skin lesions**		
		Not useful	3 (6.8)
		Somewhat useful	19 (43.2)
		Useful	22 (50.0)
**Advantages of using dermoscopy**			
	**Diagnoses melanoma in early stages**		
		Strongly agree	22 (50.0)
		Agree	19 (43.2)
		Neither agree nor disagree	3 (6.8)
		Disagree	0 (0)
	**Allows the monitoring of lesions**		
		Strongly agree	22 (50.0)
		Agree	19 (43.2)
		Neither agree nor disagree	3 (6.8)
		Disagree	0 (0)
	**Reduces the number of unnecessary biopsies or excisions**		
		Strongly agree	27 (61.4)
		Agree	14 (31.8)
		Neither agree nor disagree	3 (6.8)
		Disagree	0 (0)
	**Increases confidence in my clinical diagnoses**		
		Strongly agree	30 (68.2)
		Agree	13 (29.5)
		Neither agree nor disagree	1 (2.3)
		Disagree	0 (0)
	**Improves record-keeping**		
		Strongly agree	25 (56.8)
		Agree	14 (31.8)
		Neither agree nor disagree	4 (9.1)
		Disagree	1 (2.3)
	**Reduces patients’ anxiety**		
		Strongly agree	22 (50.0)
		Agree	12 (27.3)
		Neither agree nor disagree	10 (22.7)
		Disagree	0 (0)
	**Improves documentation for medical liabilities**		
		Strongly agree	25 (56.8)
		Agree	12 (27.3)
		Neither agree nor disagree	6 (13.6)
		Disagree	1 (2.3)
	**Increases reimbursement**		
		Strongly agree	21 (47.7)
		Agree	12 (27.3)
		Neither agree nor disagree	11 (25.0)
		Disagree	0 (0)
**Dermoscopy performance**			
	**Dermoscopy has increased the number of melanomas detected compared to naked-eye examinations**		
		Yes	30 (68.2)
		No	14 (31.8)
	**In your practice, how did the use of dermoscopy influence the number of excisions of benign lesions that you performed?**		
		Decreased the number	27 (61.4)
		Increased the number	6 (13.6)
		Did not change the number	11 (25.0)

Table 5 presents the relationship between categorical variables and the use of dermoscopy, as well as dermatologists’ training. The results established a significant association of the participants’ ages (*P*=.003), residency levels (*P*=.001), and practice centers (*P*=.004) with the use of dermoscopy among the participants. Additionally, this study established a significant association between receiving dermoscopy training and confidence levels among participating dermatology residents (*P*=.002). Furthermore, a significant association between the type of training and the type of dermoscopy use was found (*P*=.003).

**Table 5.** The association between categorical variables and dermoscopy use—association between participants’ categorical variables and the use of dermoscopy, use frequency, and training type.

Variables			Participants, n (%)			*P* value
**Gender**						.36
	Female		22 (50.0)			
	Male		22 (50.0)			
**Age (years)**						.003
	20‐25		3 (6.8)			
	26‐30		39 (88.6)			
	31‐35		1 (2.3)			
	36‐40		1 (2.3)			
**Residency level**						.001
	Residency year 1		2 (4.5)			
	Residency year 2		11 (25.0)			
	Residency year 3		9 (20.5)			
	Residency year 4		22 (50.0)			
**Device availability and cost**						.12
	Yes, I own such a device		34 (77.3)			
	It is provided in the clinic		8 (18.2)			
	No, I do not own it, nor is it provided		2 (4.5)			
**Practice center**						—^a^
	King Saud University Medical City		11 (25.0)			
	Other		33 (75.0)			
**Number of skin clinics and patients**						.45
	Less than once per month		5 (11.4)			
	1‐4 times per month		13 (29.5)			
	More than once per week		11 (25.0)			
	At least once per day		15 (34.1)			
**Type of training**						.43
	Dermoscopy training		14 (31.8)			
	Other		30 (68.2)			
**Change in excisions of benign lesions**						.22
	Yes		21 (44.7)			
	No		23 (52.3)			
**Receiving dermoscopy training**						.43
	Yes		14 (31.8)			
	No		30 (68.2)			
**Receiving dermoscopy training**						
	**Owning a dermoscope**					.13
		Yes, I own one	34 (77.3)	
		It is provided in the clinic	8 (18.2)		
		No, I do not own one, nor is one provided	2 (4.5)		
	**Degree of confidence**					.002
		Yes	36 (81.8)	
		No	8 (18.2)		
	**Type of usage**					.46
		Benign lesion	24 (54.5)	
		Pigment skin tumors	20 (45.5)		
**Dermoscopy use frequency**						
	**Lesion type**					.58
		Pigmented skin tumors	9 (20.5)	
		Nonpigmented skin tumors	13 (29.5)		
		Inflammatory skin lesions	22 (50.0)		
**Type of training**						
	**Usage type**					.003
		Dermoscope with a digital camera	2 (4.5)	
		Nonpolarized immersion-contact dermoscope	23 (52.3)		
		Polarized-light dermoscope	19 (43.2)		
	**Inflammatory skin lesion**					.57
		Yes	22 (50.0)	
		No	22 (50.0)		

a Not applicable.

## Discussion

### Principal Findings

This study aimed to assess the prevalence of dermoscopy use among dermatology residents in Riyadh, Saudi Arabia, and the need for dermoscopy training, as well as the practice’s benefits in diagnosing and treating skin diseases. The study’s sample was predominantly female. Moreover, a substantial majority of the participants were in their fourth year of residency and most of them were aged between 26 and 30 years.

This study revealed that more than half of the surveyed dermatology residents owned a dermoscope, with a considerable majority seeing a significant number of patients with cancers of all types every month. Additionally, more than half of the participants had received dermoscopy training, and a considerable proportion were pursuing academic activities provided by the residency program outside of their specialized training. The study’s findings underscore the importance of dermoscopy use and the necessity of better dermoscopy training as an invaluable tool in the earlier recognition of different dermatological diseases [25], as well as future strategic planning and enhanced dermoscopy training and practice in Saudi Arabia [26]. Our study verified that most of the participating dermatology residents used dermoscopy to manage their patients’ conditions, and they had received training on its use. A considerable proportion of the participants had used dermoscopy for 2 years, and the majority used nonpolarized immersion-contact and polarized-light dermoscopes.

These findings are consistent with those of a study conducted by Freeman et al [27] in the United States, which revealed that dermatologists apply dermoscopy in their daily routines to manage patients’ conditions and to diagnose their patients [27]. Similarly, a study conducted by Jones et al [28] on dermoscopy use as part of primary care in the United Kingdom found that dermatologists used dermoscopy to manage their patients’ conditions daily at a rate of 98.5% [28]. This study revealed that the majority of respondents used dermoscopy, with many being inspired to do so by their colleagues and mentors. However, some participants did not use dermoscopy due to the unavailability of dermoscopes in their offices and insufficient training. These findings align with those of a study by Alqahtani and AlBukhari [29] in Saudi Arabia, which identified a lack of adequate education and training among residents as a key reason for dermatologists’ reluctance to use dermoscopy. Similarly, our findings are consistent with those of a study conducted by Engasser and Warshaw [21] in the United States, which identified financial costs and lack of training as the primary reasons why dermatologists avoid using dermoscopy [30].

This study’s findings revealed that the majority of the participating dermatology residents used dermoscopy in fewer than 10% of cases involving patients with inflammatory skin lesions, in >70% of cases involving the examination of patients for pigmented skin tumors, and in <10% of cases in which patients were examined for nonpigmented skin tumors. Additionally, the majority of the participants reported that dermoscopy was useful in diagnosing melanoma, following up on melanocytic lesions, diagnosing pigmented skin tumors, and diagnosing nonpigmented skin tumors. These findings align with those of the study by Kuo et al [30], which involved dermatologists in Taiwan and noted that clinicians used dermoscopy to examine pigmented and nonpigmented lesions. This study found that the use of dermoscopy was associated with dermatologists’ increased confidence in their clinical diagnosis, that it reduced unnecessary biopsies or excisions, and that it improved record-keeping. Furthermore, the study revealed that dermoscopy use increased the number of melanomas detected compared to naked-eye examinations while also reducing the excisions of benign lesions.

This study’s demographics revealed that most of the participants were female. This preponderance can be explained by the higher proportion of female dermatologists worldwide [21,27]. In terms of age, majority of participating dermatology residents were between 26 and 30 years old. This suggests that younger dermatologists in Saudi Arabia are using dermoscopy more frequently than their older counterparts. These findings are consistent with those of a study conducted by Blum et al [31] in Germany, which reported higher dermoscopy usage rates among individuals younger than 35 years. This highlights the growing role of dermoscopy in clinical practice and the younger generation’s willingness to embrace new technologies for diagnosing and treating skin diseases.

### Limitations

Our results may be subject to several limitations. Despite the high response rate (87.5%), participants who chose to respond may have differed in their attitudes, experiences, or usage of dermoscopy compared to nonrespondents. As a cross-sectional study, it is limited by its inability to assess causal relationships. The sample size was relatively small, which could have increased the risk of sampling bias. Additionally, since the study was conducted in only one region, that is, Riyadh, its findings may not be generalizable to the entire country of Saudi Arabia. Furthermore, because the study involved an web-based questionnaire, it relied on respondents accurately documenting their responses, without the ability to verify their accuracy, which may have introduced bias.

### Conclusion

Dermoscopy has been widely adopted, with more than half of the dermatology residents in Riyadh, Saudi Arabia, using this technology. Its use is increasing among dermatology residents due to its evidence-based advantages in the early detection and diagnosis of skin diseases. The participants’ ages, residency levels, and practice centers were identified as the main factors influencing dermoscopy use in Saudi Arabia. The study also highlighted a strong willingness among young dermatologists to improve their dermoscopy knowledge and skills. Based on these findings, the study recommends that policy makers prioritize funding for dermoscopy by increasing the number of dermoscopes, as well as focusing on capacity building and training for dermatology residents.

## Abbreviations

ABCD: Asymmetrical, Border, Color, Diameter
